# Conceptualizing and Measuring Social Media Use in Health and Well-being Studies: Systematic Review

**DOI:** 10.2196/43191

**Published:** 2023-05-10

**Authors:** Mesfin Awoke Bekalu, Taisuke Sato, K Viswanath

**Affiliations:** 1 Lee Kum Sheung Center for Health and Happiness Harvard TH Chan School of Public Health Boston, MA United States; 2 Tufts University Medford, MA United States

**Keywords:** social media, health, well-being, conceptualization, measurement, technology use, screen time, computer use, usage, addict

## Abstract

**Background:**

Despite an increasing number of studies revealing both the benefits and harms of social media use on well-being, there is heterogeneity and a lack of consensus on how social media use is conceptualized, defined, and measured. Additionally, little is known whether existing literature focuses on ill-being or well-being outcomes and whether studies use theories.

**Objective:**

The main objective of this review was to examine (1) how social media use has been conceptualized and measured, (2) what health and well-being outcomes have been focused on, and (3) whether studies used theories.

**Methods:**

Studies were located through a comprehensive search strategy involving 4 steps. First, keyword searches were conducted on 6 major databases: PubMed, Web of Science, PsycINFO, Embase, ProQuest, and Annual Reviews. Second, a search was conducted on Google Scholar using the same sets of search terms, and the first 100 results were examined. Third, the reference sections of reviews identified in the first 2 rounds of searches were examined, and finally, the reference lists of the final set of papers included in the review were searched. Through a multistage screening, papers that met our inclusion criteria were analyzed.

**Results:**

The review included a total of 233 papers published between 2007 and 2020 in 51 different countries. While 66 (28%) of the studies investigated the effects of the problematic use or addiction of social media on health and well-being, 167 (72%) studied the effects of social media use as a “normal” behavior. Most of the studies used measures assessing the time users spend using social media. Most of the studies that examined the effects of problematic social media use or addiction used addiction scales. Most studies examined the association of social media use with mental illnesses such as depression, anxiety, self-esteem, and loneliness. While there are a considerable number of studies investigating physical health outcomes such as self-rated health, sleep, and sitting time or lack of physical activity, relatively a small number of studies examined social, psychological, and emotional well-being. Most of the studies 183 (79%) did not use any theory.

**Conclusions:**

Most studies conceptualized social media use as a “normal” behavior and mostly used time-spent measures, whereas a considerable number of studies conceptualized social media use as an addiction and used various addiction measures. The studies disproportionately focused on investigating the associations of social media use with negative health and well-being outcomes. The findings suggest the need for going beyond time spent to more sophisticated measurement approaches that consider the multiplicity of activities that users perform on social media platforms and the need for more theory-based studies on the association of social media use with not only negative well-being or “ill-being” but also with positive health and well-being outcomes.

## Introduction

### Background

Although concerns about the potential negative effects of new media and communication technologies are not new [1], recent political and global events such as the 2016 US election and the COVID-19 pandemic have brought intense interest in and concern about the role of social media in people’s lives and well-being. Despite a burgeoning body of research examining whether and how social media influence health and well-being [2], there is no consensus among researchers in what ways, for whom, and how social media can be harmful and helpful.

One body of work suggests that social media can offer people with a platform that overcomes distance and time barriers to connect and reconnect with others and thereby expand and strengthen their offline networks and interactions [3-7]. Ellison et al [7], for example, argue that apart from the 3 common forms of social capital (bonding, bridging, and linking), social media networks can provide people with the tools to stay in touch with their social networks after physically disconnecting from the networks and thereby benefit from a form of social capital named “maintained social capital.” In a study among university students, Ellison et al [7] found empirical support for this and argued that social media could be beneficial for young people who experience low self-esteem and low life satisfaction. In a similar vein, a study among college students found that social media use was positively related to students’ communication network heterogeneity, which, in turn, was positively associated with social capital and subjective well-being [8]. Another study by Valenzuela et al [9] also found that intense social media use, in this case, Facebook use, was positively related to individuals’ life satisfaction, among other outcomes.

Yet another body of literature also suggests that social media use could be detrimental for health and well-being. For instance, a study among young adults examining the long-term effects of Facebook use on subjective well-being found that Facebook use may undermine rather than enhance well-being [10]. Another study drawing data from a sample of adolescents and their parents in the United States found that social media use was moderately and positively associated with fear of missing out, loneliness, hyperactivity or impulsivity, anxiety, and depression [11]. Similarly, a national survey among American young adults found that individuals who used 7 to 11 social media platforms had substantially higher odds of having increased levels of depression and anxiety symptoms compared with people who used only 0-2 platforms [12]. Research has also found both negative and positive associations of social media use with health and well-being. For example, a study involving nationally representative American adults found that while routine social media use was not problematic in itself, emotional connection with the social media platforms was associated with poor self-rated physical health and well-being outcomes [4].

### Objectives of the Review

While the literature has continued to be inconclusive and contradictory, some studies, particularly reviews, have noted that there is variability or heterogeneity in how social media use was defined and measured in existing literature [13,14]. A cursory look at the literature on social media and well-being suggests that social media use could be conceptualized in at least 2 ways [4]. One conceptualization is social media use as a “normal” behavior that individuals perform for different reasons such as to connect with others, receive news, share information, or entertain themselves. This kind of use may be associated with health and well-being positively, negatively, or both. Studies conceptualizing social media use as a “normal” behavior often use time spent with social media measures or measures assessing the specific activities that users perform on social media. The second conceptualization is addiction or problematic use of social media. The literature on social media addiction draws from the broader addiction literature in defining social media addiction. Specifically, a range of addiction symptoms are used to determine social media addiction. These symptoms include mood modification (ie, changes in mood states as a result of excessive social media use), salience (ie, preoccupation with social media use), tolerance (ie, large amounts of time spent on social media), withdrawal symptoms (ie, unfavorable feelings when social media use is limited), conflict (ie, interpersonal problems as a direct result of social media use), and relapse (ie, returning to excessive use of social media after a period of abstinence) [15,16]. Studies conceptualizing and defining social media use as an addiction do not usually assess any positive health and well-being outcomes related to social media use but instead focus on the mental illnesses that are associated with the problematic use of social media. Such studies assess social media use addictions through a variety of addiction scales in the same way as other substance use and addiction problems are assessed. While these 2 conceptualizations are broad enough to capture how researchers define and measure users’ social media use experiences, the possibility of other conceptualizations cannot be ruled out. Apart from conceptualization, we also anticipate that this research might be focusing on negative health and well-being outcomes such as anxiety, depression, and insomnia rather than positive well-being outcomes such as happiness, flourishing, and positive mental health.

In undertaking this systematic review, we reasoned that one of the possible reasons for the contradictory and inconsistent findings that we see in today’s literature could be the lack of clarity and consistency in how social media use behavior is conceptualized and measured in health and well-being studies. We also sought to examine if studies use theory to guide their search to understand *why* and *how* social media use influences health and well-being. We therefore set out to review existing literature on the association of social media use with health and well-being and examine (1) how social media use has been conceptualized and measured, (2) what health and well-being outcomes have been focused on by existing literature, and (3) whether studies use theory.

### Prior Work

A number of systematic reviews and meta-analytic studies have been conducted on the link between digital media and well-being in general as well as social media use and well-being in particular. While these reviews share in common a focus on health and well-being outcomes, they considerably vary in terms of the specific digital technology, population, and study context or geography that they focus on. For example, recent reviews on digital technology use and well-being among adolescents found overall negative but small effects of digital technology use on adolescent well-being [17,18]. Another recent conceptual and empirical meta-review also found similar findings: a small negative association between social media use and mental health [19]. Several reviews have also pointed out important methodological and measurement limitations in existing literature [2,20,21]. While these and other previous reviews have analyzed and synthesized existing literature and thereby enhanced our understanding of the effects of digital media and social media on health and well-being, to our knowledge, none has examined systematically how social media use has been conceptualized and how that conceptualization influences measurement. Similarly, despite concerns about an anecdotal reference to the disproportionate focus of research on negative health or well-being effects of social media use, to our knowledge, there is no study that has systematically reviewed the existing literature and determined the proportions of studies that focus on the negative *and* positive health or well-being effects of social media use. As such, this review aims to fill this paucity of empirical evidence.

## Methods

### Search Strategy

We located relevant studies through a comprehensive search strategy that involved 4 steps [22]. First, 6 computerized databases were searched: PubMed, Web of Science, PsycINFO, Embase, ProQuest, and Annual Reviews in April 2020. For the search, social media-related terms were paired with health- and well-being–related terms. Specifically, we combined, using Boolean operators, 2 social media–related terms (“social media use” and “social networking sites”) and 8 most popular social media platforms (Facebook, Instagram, YouTube, Twitter, WhatsApp, TikTok, Snapchat, and WeChat or Weixin) with different health- and well-being–related terms, such as “health,” “physical health,” “self-rated health,” “mental health,” “positive mental health,” “happiness,” “well-being,” “subjective well-being,” “psychological well-being,” “social well-being,” “depression,” “distress,” “attention-deficit syndrome,” “fear,” “anxiety,” “sleep,” “hyperactivity,” and “schizophrenia.” Second, we searched Google Scholar using the same sets of search terms as above and examined the first 100 results. Third, the reference sections of reviews on social media use and health or well-being identified in the first 2 rounds of searches were examined. Finally, the reference lists of the final set of papers included in our review were searched. Our inclusion and exclusion criteria are presented in Textbox 1.

**Figure 1:**
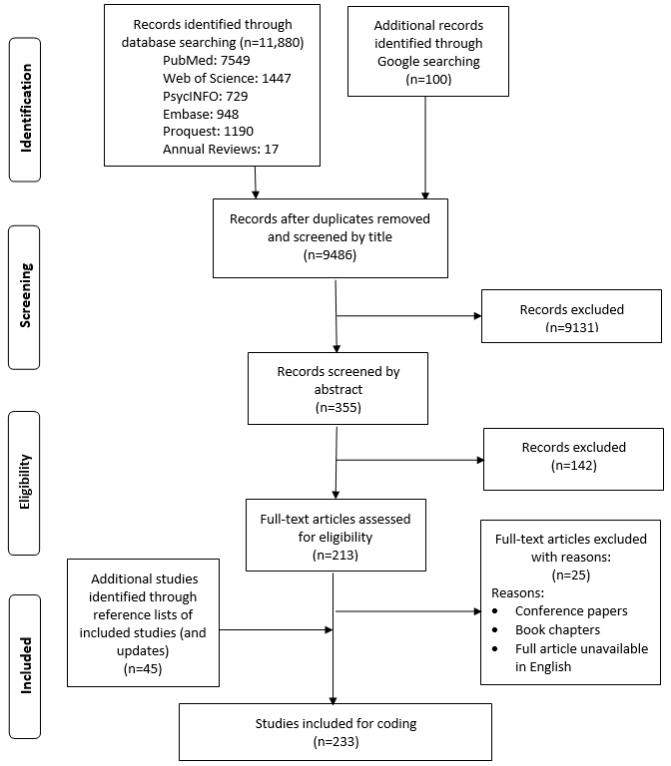
Flow diagram.

The initial search from the 6 databases and Google Scholar yielded a total of 11,880 references. After removing duplicates, 9486 references remained. Two trained reviewers screened study titles independently and reduced the number to 355 by removing studies that did not meet our inclusion criteria. They then reviewed abstracts and reduced the number further to 213, and then, located the full text of the 213 papers and screened, noting reasons for study exclusion. The full-text screening resulted in the discarding of 25 papers for different reasons (see Figure 1). During this stage, the reviewers examined the reference lists of several review papers as well as those of the 213 papers and identified 45 eligible studies. This process identified a total of 233 papers for the systematic review (Figure 1). At all stages of the review, if the 2 reviewers differed in their determination about any of the papers, they consulted a third reviewer to resolve the discrepancy and make a final determination.

**Inclusion and exclusion criteria.**

**Paper type**

- Inclusion criteria
  Reports the “unidirectional” link or association between social media use and health- and well-being–related outcomes
- Exclusion criteria
  Reports on the effects of different health conditions (eg, depression or loneliness) on social media use behaviors
  Reports on the use of social media as a tool, channel, or platform to deliver health promotion and disease prevention interventions
  Reported on social media scale development

**Language**

- Inclusion criteria
  Available in English
- Exclusion criteria
  Not available in English

### Paper Coding

We developed a coding guide and coding categories based on relevant literature. The guide outlined the rubrics for extracting the required information from the papers and provided operational definitions of the constructs to be coded. The coding categories included 11 elements of interest: study setting, study design, sampling, sample population characteristics, use of theory, type of social media studied, type of health and well-being studied, conceptualization of social media use, measures used to assess social media use, type of association reported, and type of effect or association moderators reported.

Two trained coders coded the papers. Intercoder reliability was assessed by double coding 70 (30% of the final set) papers. Krippendorff α [23] ranged from a low of .70 to a high of .97. All discrepancies and issues that arose during the coding process were resolved through discussion between the 2 coders and a third reviewer.

## Results

### Overall Description of Reviewed Papers

The 233 studies were published between 2007 and 2020, with a median publication year of 2018. The number of studies increased over time, with 192 (82%) published after 2016, a total of 37 (16%) between 2011 and 2015, and only 4 (less than 2%) between 2007 and 2010. The studies had a cumulative sample size of N=1,258,298 and were conducted in 51 different countries. Most studies, 77 out of 233 (33%) were conducted in the United States; followed by the United Kingdom 22 studies (9%); Australia, China, and the Netherlands, 11 studies each (4.7%); and Canada, 8 studies (3.4%). In terms of income, 42 studies (about 18%) were conducted in low- and middle-income countries. The majority of studies were conducted in a single country, but 5 studies were conducted across multiple countries.

Most studies, 173 out of 233 (74%), used cross-sectional study design, followed by 49 longitudinal (21%) and 10 experimental (4%) study designs. Only 3 studies (about 1% of the total) used a qualitative study design. Consistent with previous reviews [2], most of the studies (more than 50%) that used experimental design were conducted in recent years (2019 and 2020). The majority of the studies, 213 out of 233 (91%), used convenience sampling method, with the remaining 20 (9%) using probability-based sampling methods.

While the majority of the studies, 213 out of 233 (91%), involved both sexes, 13 (5%) and 6 (2.5%) studies involved only female or male participants, respectively. In terms of age, the majority of the studies, 161 out of 233 (69%), studied young population groups (<24 years), whereas 72 studies (31%) involved general adult population (18 years and older in most cases). Only 3 studies (about 1%) focused on older population groups (>65 years).

Most of the studies, 136 out of 233 (52%), investigated general social media or social networking sites, whereas 84 studies (32%) studied Facebook. Apart from that, Instagram (n=20, 8%), Twitter (n=13, 5%), and Snapchat (n=6, 2%) are the most commonly studied social media platforms.

### Conceptualization and Measurement of Social Media Use

Most of the studies, 167 out of 233 (approximately 72%), studied social media use as a “normal” behavior, whereas the remaining 66 studies (28%) studied the effects of problematic social media use or addiction.

To measure social media use, most of the studies, 99 out of 233 (28%), used duration of use or length of time users spend on social media, while 88 (25%) studies used frequency of use or how often users login on to social media. A considerable number of studies (n=65, 18.16%) that examined the effects of problematic social media use or addiction used addiction scales.

### Type of Health and Well-being Outcomes Studied

A majority of the studies (155/233, 48%) examined the association of social media use with mental illnesses such as depression, anxiety, self-esteem, and loneliness. A small number of studies (55/233, 17%) studied physical health outcomes such as self-rated health, sleep, and sitting time or lack of physical activity. Very small numbers of studies examined social (n=39, 12%), psychological (n=38, 11.8%), and emotional (n=34, 10.6%) well-being.

### Type of Effect or Association Reported

Most of the studies (142/233, 60%) reported the negative or health or well-being–compromising effects of social media use, whereas 50 studies (21%) reported mixed outcomes—positive effects on some health or well-being outcomes and negative effects on others. Only 23 out of 233 studies (10%) reported positive or health or well-being–enhancing effects, whereas about the same number of studies (n=20, 9%) reported no association or effect of social media use with or on health or well-being outcomes.

It should be noted that 57 (40%) of the studies that reported negative associations or effects conceptualized social media use as addiction and used one or another form of addiction scale. Additionally, 87 (52%) out of the total of 167 studies that conceptualized social media use as a “normal” social behavior and almost all of the studies that conceptualized social media use as addiction reported either negative or no associations or effects. Similarly, compared with 20 studies (12%) that conceptualized social media use as a “normal” social behavior, only 1 study that conceptualized social media use as an addiction reported a positive association or effect.

### Use of Theory

While most of the studies (183/233, 79%) did not use any theories, only 50 studies (21%) used theories such as social comparison, social displacement, and gratification theories.

## Discussion

### Principal Findings

The purpose of this study was to systematically review existing literature and examine (1) how social media use has been conceptualized and measured, (2) what health and well-being outcomes have been focused on, and (3) whether studies drew on social and behavioral theories in studying social media effects. The review found that most studies conceptualized social media use as a “normal” behavior and mostly used time-spent measures, whereas a considerable number of studies conceptualized social media use as an addiction and used various addiction measures. Most studies disproportionately focused on investigating the associations of social media use with negative health and well-being outcomes and did not use and draw on any social or behavioral theories.

We examined 233 peer-reviewed papers coming from 51 countries with a cumulative sample size of 1,258,298. We found the number of studies on the association of social media with health and well-being increased over time, suggesting a growing interest in and concern about whether and how social media use affects health and well-being. Consistent with previous reviews [2], results of this review indicated that most of the studies used cross-sectional study design, while relatively low and very low numbers of studies used longitudinal and experimental designs. More than half the studies that used experimental design were conducted in recent years (2019 and 2020). Of note, only 3 (1%) of the reviewed studies used a qualitative study design. It should be noted that although the number of studies has generally increased over time, there is still a lack of studies using rigorous study designs (eg, longitudinal and experimental) which raise questions about causality between social media use and health or well-being. Beyond structured interviews, surveys, and longitudinal and experimental studies using self-reported closed- and open-ended questions, qualitative approaches investigating social media content are important to elucidate the complex link between social media use and well-being. In other words, existing research has barely studied exposure to content on social media through more in-depth qualitative methods. Moreover, the review found that the majority of the studies used a convenience sampling method, while 20 (9%) used a probability-based sampling method, suggesting the potential of biases in the conclusions drawn by most of the existing literature on social media and well-being.

Interestingly, the review found that the majority of the studies 213 (91%) involved both sexes, while a small percentage involved only female or male participants, suggesting the absence of bias in terms of gender. In terms of age, the majority of the studies focused on young population groups (<24 years), whereas relatively a small number of studies involved general adult population (18 years and older in most cases), and only about 3 (1%) of the studies focused on older population groups (>65 years). It should be noted that despite the fact that social media use has increasingly become popular across all age groups [24], most studies have focused on adolescent and young adult samples. Nevertheless, this focus on young population groups may also be justified in view of recent studies suggesting that younger age represents a window of developmental sensitivity to social media use [25].

Results indicated that most of the studies investigated the effects or associations of general social media or social networking sites with health and well-being. A large number of studies also investigated the effects of using Facebook on health and well-being. As acknowledged by previous studies [2], the focus of this research on the aggregate effects of different social media platforms remains a concern. Obviously, despite some common features, different social media platforms have different features and affordances (eg, synchronicity, quantifiability, publicness, persistence, and visibility) that would lead to differences in user engagement and experience and associated health- and well-being–related outcomes.

With the above overall description of the reviewed studies, we now focus on how social media use has been conceptualized and measured in existing health and well-being studies. Results indicated that most of the studies conceptualized social media use as a “normal” behavior, whereas a considerable number of studies focused on the effects of problematic social media use or addiction. These conceptualizations are important for at least 2 reasons. First, studies conceptualizing social media use as a “normal” behavior that people perform for different reasons are more likely to adopt a “neutral” stance and hypothesize both positive and negative effects of social media use on users’ health and well-being. On the contrary, studies conceptualizing social media use as an addiction focus on the detrimental effects of the addictive behavior. Second, in terms of measurement, the review revealed that while studies conceptualizing social media use as a “normal” behavior used a wide variety of measures including time-spent measures (duration and frequency of use) to assess both negative and positive effects of social media use, studies focusing on addiction or problematic use of social media use used “addiction scales” that would not enable them to capture any positive effects of social media use on health and well-being.

The review indicated that the majority of the studies investigated the effects of social media use on ill-being—mental illnesses such as depression, anxiety, self-esteem, and loneliness, whereas only a small number of studies investigated social, psychological, and emotional well-being. While the focus on ill-being may seem to be consistent with the dominant pathogenesis paradigm that has characterized the medical field for a century [26], it stands in stark contrast to the increasing recognition of the role of positive health and salutogenesis [27,28] in recent years. It should be noted that because the positive and negative effects of social media use on health and well-being are not necessarily complementary, an overwhelming focus on their detrimental effects on mental health may not necessarily provide a complete picture of the association between social media use and well-being.

Regarding the type of effect or association reported, most of the studies reported the negative or well-being–compromising effects of social media use, whereas a considerable number of studies reported mixed outcomes—positive effects on some well-being outcomes and negative effects on others. Only a small number of studies reported positive or well-being–enhancing effects, whereas about the same number of studies reported no association or effect of social media use with or on health or well-being outcomes. It should be noted that about 57 (40%) of the studies that reported negative effects conceptualized social media use as addiction and used one or another form of addiction scale.

The review found that while a considerable number of studies used theories such as social comparison, social displacement, and gratification theories, most of the studies did not use any theories. This could be related to the fact that social media researchers come from different disciplines or scientific fields, while interdisciplinarity is desirable for many reasons, it may make the development of unified conceptual and theoretical frameworks difficult for the study of social media. Researchers have also voiced concern about a potential “jingle-jangle” problem—a situation in which different terms are used to refer to the same process—as an unintended byproduct of the interdisciplinary nature of social media research [2].

### Limitations

The review has some limitations. The first limitation is the fact that papers published in languages other than English were not included. However, given that 98% of publications in science are written in English [29], we anticipate that most of the studies on social media use and well-being would be in English rendering considerable generalizability to our conclusions. Second, given our focus on the “effects” of social media use on health and well-being outcomes and how it has been conceptualized and measured in existing health and well-being studies, papers that reported on the effects of different health conditions (eg, depression or loneliness) on social media use behaviors were not included. Third, review papers and studies that reported on the use of social media as a tool, channel, or platform to deliver health promotion and disease prevention interventions as well as studies that reported on social media scale development were not included.

### Conclusions

In the absence of a clear understanding of how studies conceptualize and measure the social media use behavior, it is difficult to draw any conclusions about their effects on health and well-being. Many observers including researchers have voiced concern about the detrimental effects of social media use on health and well-being. In most cases, such concerns are backed by one or more research studies, although how the studies have conceptualized, defined, and measured are often not taken into account. For example, a study conceptualizing social media use as an addiction and setting out to find out the effects of problematic use of social media will hardly report any positive or desirable outcomes. In this review, for example, almost all of the studies that conceptualized social media use as an addiction reported either negative or no effects of social media use on positive health and well-being outcomes. As such, any conclusions about the effects of social media use on health and well-being need to take these 2 broad types of conceptualizations into account. The review has confirmed the concern of many researchers that there is a substantial understudy of the positive effects of social media use on health and well-being. This has been demonstrated by the disproportionately large number of studies that investigated “ill-being” rather than “well-being.” Again, in order to have a complete picture of the harms and benefits of social media use, there is a need to refocus current and future research on both ill-being and well-being outcomes. Last but not least, the review has revealed the lack of theory in the majority of the studies. Given the usefulness of theory to guide researchers in their search to understand why and how social media use influences health and well-being, the lack of theory in current social media literature should be a concern. In scientific research, theories are useful to inform research design, the development of measures, and the interpretation of findings. Atheoretical studies suffer from a lack of specificity and a neglect of the underlying processes and mechanisms that might explain why social media use may lead to harmful or beneficial outcomes.

## Data Availability

The data sets generated and analyzed during this study are available from the corresponding author upon reasonable request.
